# Molecular Adaptations in the Rat Dorsal Striatum and Hippocampus Following Abstinence-Induced Incubation of Drug Seeking After Escalated Oxycodone Self-Administration

**DOI:** 10.1007/s12035-018-1318-z

**Published:** 2018-08-28

**Authors:** Christopher A. Blackwood, Reece Hoerle, Michael Leary, Jennifer Schroeder, Martin O. Job, Michael T. McCoy, Bruce Ladenheim, Subramaniam Jayanthi, Jean Lud Cadet

**Affiliations:** Molecular Neuropsychiatry Research Branch, NIH/NIDA Intramural Research Program, 251 Bayview Boulevard, Baltimore, MD 21224 USA

**Keywords:** Oxycodone, Opioid receptors, Protein, mRNA, Incubation, Dorsal striatum, Hippocampus

## Abstract

Repeated exposure to the opioid agonist, oxycodone, can lead to addiction. Here, we sought to identify potential neurobiological consequences of withdrawal from escalated and non-escalated oxycodone self-administration in rats. To reach these goals, we used short-access (ShA) (3 h) and long-access (LgA) (9 h) exposure to oxycodone self-administration followed by protracted forced abstinence. After 31 days of withdrawal, we quantified mRNA and protein levels of opioid receptors in the rat dorsal striatum and hippocampus. Rats in the LgA, but not the ShA, group exhibited escalation of oxycodone SA, with distinction of two behavioral phenotypes of relatively lower (LgA-L) and higher (LgA-H) oxycodone takers. Both LgA, but not ShA, phenotypes showed time-dependent increases in oxycodone seeking during the 31 days of forced abstinence. Rats from both LgA-L and LgA-H groups also exhibited decreased levels of striatal mu opioid receptor protein levels in comparison to saline and ShA rats. In contrast, mu opioid receptor mRNA expression was increased in the dorsal striatum of LgA-H rats. Moreover, hippocampal mu and kappa receptor protein levels were both increased in the LgA-H phenotype. Nevertheless, hippocampal mu receptor mRNA levels were decreased in the two LgA groups whereas kappa receptor mRNA expression was decreased in ShA and LgA oxycodone groups. Decreases in striatal mu opioid receptor protein expression in the LgA rats may serve as substrates for relapse to drug seeking because these changes occur in rats that showed incubation of oxycodone seeking.

**Electronic supplementary material:**

The online version of this article (10.1007/s12035-018-1318-z) contains supplementary material, which is available to authorized users.

## Introduction

Addiction to opioid agonists is a public health menace [1, 2]. This is related to the over-prescription and illicit use of these agents, including oxycodone, for the treatment of various pain syndromes [3, 4]. Patients treated with these drugs usually increase their intake of opioids as they become tolerant to the clinical effects of the drugs or suffer from withdrawal signs and symptoms and go through repeated relapses [5]. Unfortunately, efforts to treat some of these clinical manifestations of abuse have not always been met with a great degree of success and may have complicated the clinical history of oxycodone by promoting drug craving, one of the driving forces of repeated relapses marred by compulsive drug seeking and uncontrollable use [5].

Attempts to treat opioid drug craving have targeted classical opioid receptors [5, 6]. These include mu (OPRM1), delta (OPRD1), and kappa (OPRK1) opioid receptors [7–9]. Opioid receptors are members of the G protein coupled receptors family that can form homo- and heterodimeric complexes and signal via kinase cascades [10, 11]. These receptors may also play relevant and diverse roles in the signs and symptoms of opioid withdrawal. For example, Src-dependent phosphorylation of mu receptors is a prerequisite for naloxone-induced withdrawal in mice treated chronically with morphine [12] while constitutive mu receptor activation is also enhanced in the ventral tegmental area (VTA) of animals undergoing morphine withdrawal [13]. Moreover, antagonism of kappa receptors with nor-binaltorphine (nor-BNI) was reported to reduce morphine withdrawal symptoms in rats [14]. Thus, in order to develop more logical therapeutic approaches against addiction to opioids and other drugs, it is essential to understand the biochemical and molecular neurobiology of drug seeking after long periods of abstinence from drug self-administration (SA) [4, 15].

In the case of oxycodone addiction, it has been shown that animals, given various lengths of access to the drug during SA experiments, will show various degrees of escalation of oxycodone intake and exhibit compulsive drug seeking [16–18], behaviors that may be secondary to neuroadaptive changes in striatum-dependent habitual behaviors [19–21] and/or hippocampus-mediated mnemonic properties [22–25]. These circuits and their potential roles in addictive processes have been reviewed extensively [26]. At present, however, very little is known about the biochemical and molecular consequences of long-term oxycodone exposure to the brain. In a first attempt to fill some of these gaps, we have used both short- and long-access oxycodone self-administration to identify potential outcomes of forced abstinence on drug-seeking behaviors. We also wanted to identify the effects of withdrawal from oxycodone self-administration on biochemical and molecular markers of opioid circuitries in the rat dorsal striatum and hippocampus in animals that showed variable cue-induced behavioral responses. To achieve these goals, we have measured the mRNA and protein expression of the three opioid receptors in these two brain regions that express the three receptors [27–29]. Parenthetically, oxycodone has also been reported to interact with the three relevant opioid receptor proteins [30–33].

## Materials and Methods

### Subjects

Male Sprague Dawley rats, (Charles River, Raleigh, NC, USA) weighing 350–400 g before surgery, were used in our experiments. Rats were maintained on a 12-h reversed light/dark cycle with food and water available ad libitum. All procedures followed the guidelines outlined in the National Institutes of Health (NIH) Guide for the Care and Use of Laboratory Animals (eighth Edition, https://guide-for-the-care-and-use-of-laboratory-animals.pdf) and were approved by the local NIDA (National Institute of Drug Abuse) Animal Care and Use Committee.

### Intravenous Surgery

Surgical implantations of intravenous catheter were done as previously described [34]. Briefly, we anesthetized the rats with an intraperitoneal injection of ketamine (50 mg/kg) and xylazine (5 mg/kg) and inserted polyurethane catheters (SAI Infusion Technologies, Lake Villa, IL) into the jugular vein. One end of the catheters was in the jugular vein while the other end was attached to modified 22-gauge cannulas that were mounted to the rats’ backs with dental cement to serve as catheter externalized infusion ports. The catheter infusion ports were closed using dust caps (PlasticOne, Roanoke, VA). We used subcutaneous injections of buprenorphine (0.1 mg/kg) after surgery to relieve pain and allowed the rats to recover for 5–7 days before oxycodone self-administration (SA) training. Thereafter, the catheters were flushed every 48 h with gentamicin (0.05 mg/kg, Henry Schein, Melville, NY) and sterile saline to maintain patency.

### Apparatus

Rats were trained in SA chambers located inside sound-attenuated cabinets and controlled by a Med Associates System (Med Associates, St Albans, VT). Each chamber was equipped with two levers located 8.5 cm above the grid floor. Presses on the retractable active lever activated the infusion pump and tone-light cue. Presses on the inactive lever had no reinforced consequences. The catheters were connected to modified cannulas (Plastics One, Roanoke, VA) connected to a fluid swivel (Instech Plymouth, PA) via polyethylene-50 tubing that was protected by a metal spring.

### Training Phase

**Fig. 1:**
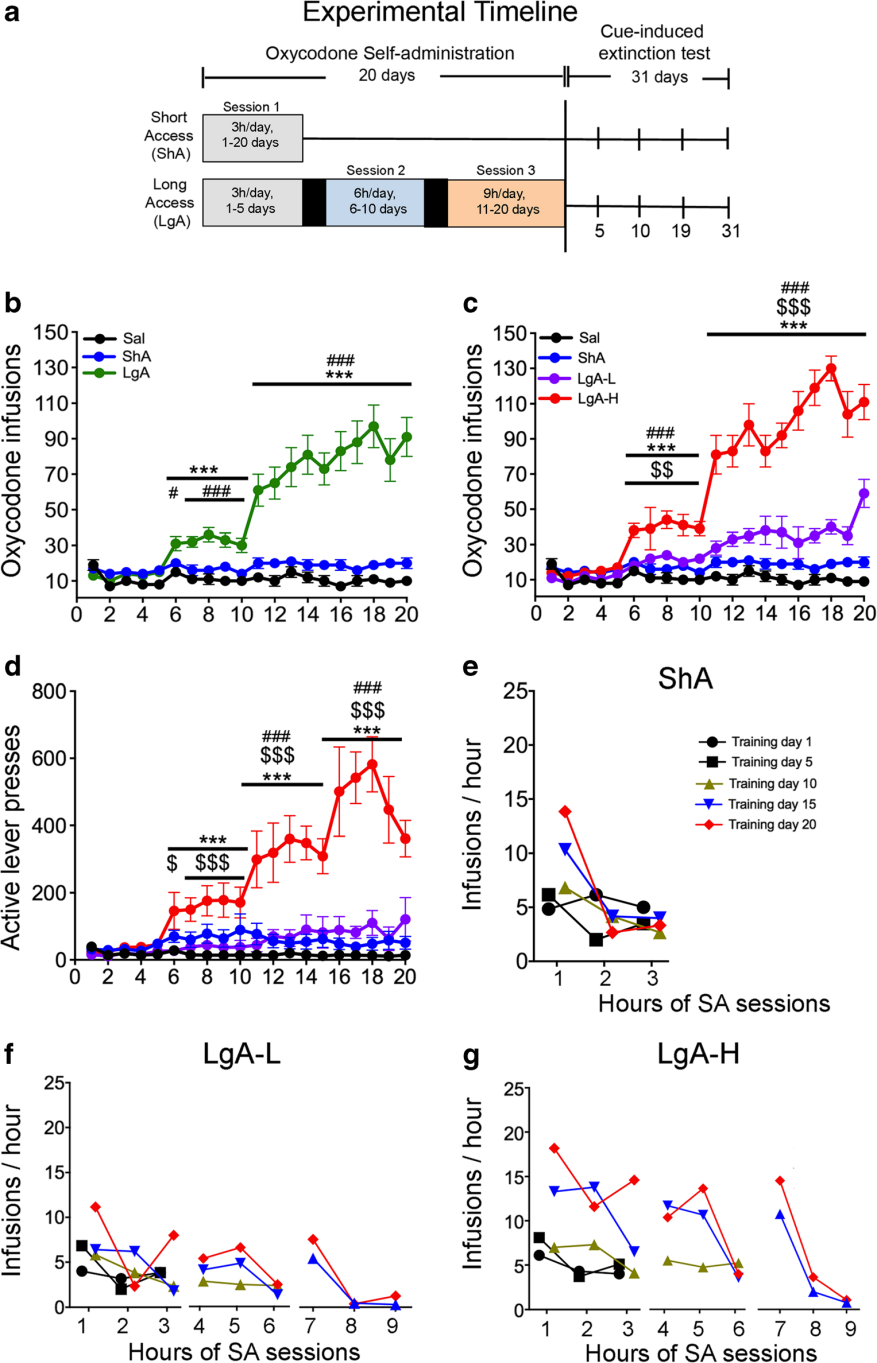
Rats with long access to oxycodone excalate their drug intake during self-administration. **a** Experimental timeline of oxycodone self-administration (SA) experiments. Saline (*n* = 8), short-access (ShA) (*n* = 15) rats and long-access (LgA) (*n* = 15) rats were trained as described in the text. After the last day of SA, rats underwent cue-induced extinction test for 3 h on withdrawal days 5, 10, 19, and 31. **b** LgA rats escalate their oxycodone intake during the training sessions. **c** LgA rats show two distinct intake phenotypes, high (LgA-H) (*n* = 9) and low (LgA-L) (*n* = 6) drug takers during the escalation stage. **d** LgA-H rats show higher active lever presses in comparison to all drug-taking phenotypes. **e**–**g** ShA, LgA-L, and LgA-H rats take more drug during first hours of the three 3-h daily sessions. Individual statistics are described in Supplemental Figs. S1, S2, and S3. The values represent means ± SEM. Key to statistics: *, **, *** = *p* < 0.05, 0.01, 0.001, respectively, in comparison to saline rats; ^#^, ^###^ = *p* < 0.05, 0.001, respectively, in comparison to ShA rats; ^$^, ^$$^, ^$$$^ = *p* < 0.05, 0.01, 0.001, respectively, in comparison to LgA-L rats

Rats (*n* = 38) were housed in Med Associates SA chambers and were randomly assigned to either saline (Sal) (*n* = 8) or oxycodone (*n* = 30) conditions. Short-access (ShA) rats were trained to self-administer oxycodone-HCL (NIDA Pharmacy, Baltimore, MD) for only one 3-h session (*n* = 15) throughout the duration of the study (days 1–20). Long-access (LgA) rats were trained to self-administer for three sessions: one 3-h session during days 1–5, followed by two 3-h sessions during days 8–14, and then for three 3-h sessions during the rest of the study (days 15–20) (see Fig. 1a for a descriptive rendering of the study time course). For the LgA group, the 3-h sessions were separated by 30-min intervals from day 6 to day 20; during these interludes, rats remained in operant chambers but had no access to the levers to press for oxycodone (Fig. 1a). Lever presses were reinforced using a fixed ratio-1 with a 20-s timeout accompanied by a 5-s compound tone-light cue. We used a scheduling pattern of 5 days of drug SA and 2 days off to control for weight loss, a common side effect of oxycodone intake in laboratory animals [18]. Rats self-administered oxycodone at a dose of 0.1 mg/kg per infusion over 3.5 s (0.1 ml per infusion). The house light was turned off and the active lever retracted at the end of the 3-h session. After training day 21, rats were returned to the animal vivarium and individually housed with no access to oxycodone. Intravenous catheters exit ports were closed using dust caps, and rats had access to home-cage food and water ad libitum.

### Withdrawal Phase

Cue-induced drug craving was then assessed at days 5, 10, 19, and 31 of withdrawal under extinction conditions. During this phase, rats were brought back to their corresponding SA chamber on the morning of each test. However, no oxycodone infusions were available. Each test consisted of a 3-h session during which presses on the drug-associated lever resulted in contingent presentations of the tone-light cues that were previously paired to oxycodone infusions during the training phase.

### Tissue Collection

We euthanized rats 24 h after the last withdrawal day (WD31). Brains were removed from skulls and the dorsal striata and hippocampi were dissected, and snap frozen on dry ice. Dorsal striatum and hippocampi were visually dissected using distinguishable anatomical structures (corpus callosum and optical trac). Dissections were guided using coordinates (Dorsal striatum: A/P +2 to − 2 mm bregma, mediolateral ± 2 to 5 mm, D/V − 3 to − 6 mm and hippocampi: A/P − 5 to −7 mm bregma, mediolateral ± to 6 mm, D/V − 2 to −8 mm) according to Paxinos and Watson, 1998. Tissues were subsequently used for western blotting and quantitative RT-PCR (qPCR) analyses.

### Quantitative RT-PCR

Total RNA was isolated from one hemisphere of the dorsal striatum and hippocampus using RNeasy Mini Kit (Qiagen, Valencia, CA). Total RNA (0.5 μg) was reverse-transcribed (RT) with oligo dT primers using Advantage RT-for-PCR kit (Clontech, Mountain View, CA). RT-qPCR was performed as previously described [34] with Roche LightCycler 480 II (Roche Diagnostics, Indianapolis, IN) using iQ SYBR Green Supermix (Bio-Rad, Hercules, CA). For all RT-qPCR experiments, individual data were normalized using the corresponding *B2M*, *Clathrin*, or *GAPDH* mRNA levels. The results are reported as fold changes calculated as the ratios of normalized gene expression data for oxycodone SA groups compared to the saline group. All the quantitative data are presented as means ± SEM. Primer sequences for *Oprm1*, *Oprd1*, *Oprk1*, *B2M*, *Clathrin*, and *GAPDH* are listed in Table 1. Custom-designed primers were manufactured and HPLC-purified by the Synthesis and Sequence Facility of the Johns Hopkins University (Baltimore, MD).

**Table 1:** List of RT-qPCR primers

Gene name	Forward	Reverse
OPRM1	CATATTCACCCTCTGCAC	TTACAGGCAGACCGATG
OPRD1	TTACAGGCAGACCGATG	ATGTTTGGAATCGTCCGGTACA
OPRK1	TCTAGCTATTACTTCTGCATTG	TGTGTTTCTAACTCTGTTTGT
B2M	GATCTTTCTGGTGCTTGT	AGCTCAATTTCTATTTGAGGT
Clathrin	AAG TAT CCG TAA GTC GAG	GGGGTTAAAGTCACAGAG
GADPH	CCTTCTCTTGTGACAAAGTG	CCCATTTGATGTTAGCGG

### Western Blotting

Tissues were homogenized using 10 mM Tris HCl, 150 mM NaCl, pH 7.5 in the presence of 1% Nonidate P-40 (NP-40), protein, and phosphatase inhibitor cocktails (Sigma, St. Louis, MO). Total protein concentrations were measured using BCA assay (ThermoFisher Scientific, Waltham, MA). Ten to 30 μg of soluble protein lysate were prepared in solutions that contained 1× NuPage LDS Sample Buffer (ThermoFisher Scientific, Waltham, MA), and 1% B-Mercaptoethanol. Samples were then boiled and resolved using NuPage 10% Bis-Tris Protein Gels (ThermoFisher Scientific, Waltham, MA). Protein were electrophoretically transferred onto Trans-Blot® Turbo™ Midi Nitrocellulose membranes using the Trans-Blot® Turbo™ system (Bio-Rad, Hercules, CA). Membrane blocking, antibody incubations, and chemiluminescence reactions were performed according to the protocol described by manufacturer. Primary rabbit polyclonal antibodies including anti-OPRM1 (1:5000, ab17934), anti-OPRK1 (1:10000, ab183825), anti-OPRD1 (1:1000, ab176324), and anti-Cyclophilin B (CYPB) (1:10000, ab16045) were purchased from Abcam (Cambridge, MA). α-Tubulin (α-Tub) mouse monoclonal antibody was from Sigma-Aldrich (St. Louis, MO) (1:10000, T6074). There are previous publications with the antibodies against opioid receptors [35]. In our study, the antibodies revealed bands at the expected molecular weights for the proteins. Secondary antibodies used were goat anti-rabbit (1:500, Sc-2004) and goat anti-mouse (1:1000, Sc-2005) conjugated HRP purchased from Santa Cruz Biotechnology (Dallas, TX). Following secondary antibody incubation, ECL clarity (Bio-Rad, Hercules, CA) was used to detect gel bands on ChemiDoc Touch Imaging System (Bio-Rad, Hercules, CA), and intensities were quantified with Image Lab 6.0 version (Bio-Rad, Hercules, CA) software.

### Statistical Analyses

Behavioral data were analyzed using repeated-measures analysis of variance (ANOVA). Dependent variables were the number of oxycodone infusions, active lever presses, and inactive lever presses on training days. Independent variables were between-subject factor reward types (saline, ShA, LgA-L, LgA-H), within-subject factor SA day (training days 1–20), and their interactions. If the main effects were significant (*p* < 0.05), Bonferroni post hoc tests were used to compare reward types on each training day while maintaining an overall type I error rate of 0.05. Extinction data were also analyzed using ANOVA: the dependent variable was active lever presses on extinction days 5, 10, 19, and 31 and the independent variables were group (saline, ShA, LgA-L, LgA-H), extinction day (5, 10, 19, 31) and their interaction. A statistically significant interaction was followed by Bonferroni post hoc tests comparing the same extinction day across groups. Fisher’s PLSD post hoc tests compared extinction days within each group. Biochemical data were also analyzed using one-way ANOVA followed by the Fisher’s PLSD post hoc test if the main effect was significant. Statistical significance for all hypothesis tests was set at *p* < 0.05. Behavioral data were analyzed with SPSS version 24 (IBM, Armonk, NY), and biochemical data were analyzed using StatView version 4.0 (SAS, Cary, NC). Non-linear and linear regression analyses were performed to see if there were any correlations between the effects of oxycodone and extinction days or protein expression. The slopes of all the regression lines were calculated using one-way ANOVA.

## Results

### Rats Exposed to LgA Oxycodone Self-Administration Escalate Their Drug Intake over Time

Figure 1 shows the results of the behavioral studies. ShA rats (*n* = 15) were trained for 3 h for 20 days whereas LgA rats (*n* = 15) were trained for 3 h for 1–5 days, 6 h for 6–10 days, and 9 h for 11–20 days (Fig. 1a). The repeated-measures ANOVA for reward earned included the between-subject factor group (Sal, ShA, LgA) and the within-subject factor of SA day SA (training days 1–20), and the group × day interaction. This analysis showed statistically significant effects for group (*F*_(2,35)_ = 32.26, *p* < 0.0001), day (*F*_(19,17)_ = 11.05, *p* < 0.0001), and group × day interaction (*F*_(38, 36)_ = 3.03, *p* = 0.001). A similar model comparing LgA rats to saline rats found statistically significant effects of group (*F*_(1,21)_ = 32.52, *p* < 0.0001), non-significant effect of day (*F*_(14,8)_ = 1.41, *p* = 0.32), and group × day interaction (*F*_(14, 8)_ = 5.75, *p* = 0.009), indicating that LgA rats increased their oxycodone intake substantially after training day 5 compared to saline rats. An analogous model comparing ShA rats to saline rats found statistically significant effects of group (*F*_(1,21)_ = 12.42, *p* = 0.002) and day (*F*_(14,8)_ = 11.90, *p* = 0.001) but no significant group × day interaction (*F*_(14, 8)_ = 1.75, *p* = 0.21) (Fig. 1b).

Close inspection of the behavioral data revealed that LgA animals consisted of two SA phenotypes: groups of relatively lower (LgA-L) (*n* = 6) or higher (LgA-H) (*n* = 9) oxycodone takers (Fig. 1c, d). The LgA-L phenotype consisted of rats that took less than 50 infusions per day while the LgA-H took more than 50 infusions (Fig. 1c). LgA-H rats also showed greater escalation of their oxycodone intake than the LgA-L rats (Fig. 1c). Figure 1d illustrates the differences in active lever presses between the four groups and demonstrates that escalation of oxycodone intake was accompanied with a substantial increase in active lever presses after day 6 in the LgA-H (*F*_(13, 208)_ = 4.17, *p* < 0.001) but not in the ShA (*F*_(13, 273)_ = 0.59, *p* = 0.630) rats compared to saline rats.

We also sought to determine if patterns of hourly drug consumption might also differ between the ShA, LgA-L, and LgA-H (Figs. 1e–g) groups. We found that ShA rats took most of the oxycodone infusions during the first hour of their 3-h exposure that we used during the 20 sessions (Fig. 1e and Supplemental Figure 1S). Similarly, both LgA-L and LgA-H groups took most of their oxycodone infusions during the first hour of each 3-h session of daily training during the 20 days of training (Fig. 1f, g, and Supplemental Figures S2 and S3). These results suggest that even animals that ended up taking small amounts of oxycodone still exhibited a binge pattern of drug-taking behavior by taking most of their infusions during the first hour of oxycodone exposure. Statistical analyses for the hourly patterns of oxycodone infusions are further detailed in Supplemental Figures S1-S3.

### LgA, but Not ShA, Rats Exhibit Incubation of Oxycodone Seeking During Prolonged Forced Abstinence

**Fig. 2:**
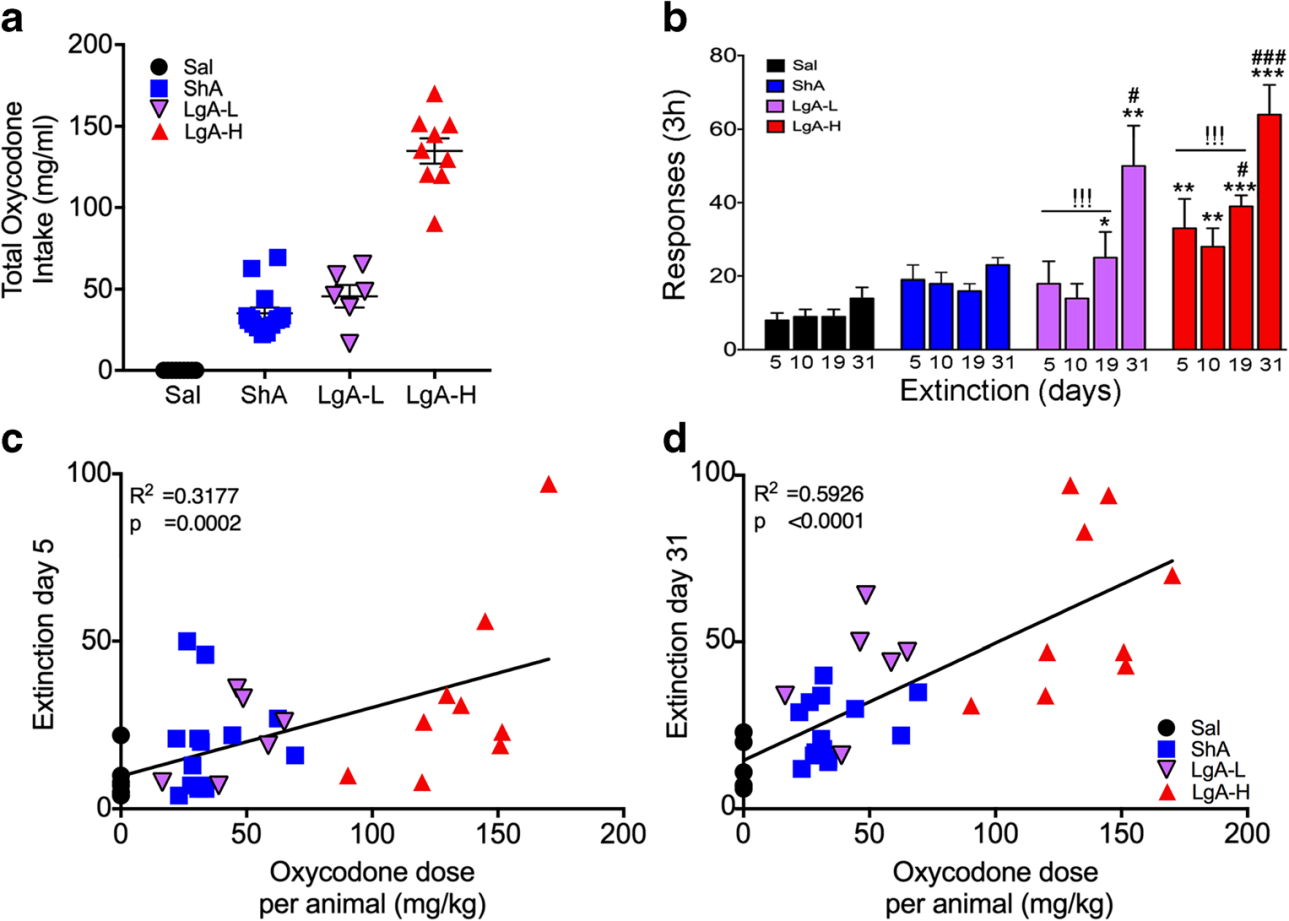
Extinction of oxycodone causes time-dependent incubation of oxycodone seeking in long-access rats. **a** Total oxycodone intake in rats during the training phase. **b** LgA-L and LgA-H rats show significant increases in lever pressing at withdrawal day 31 compared to other groups tested on the same day. Individual oxycodone intake is positively correlated to number of lever presses at **c** withdrawal day 5 and **d** day 31. The values represent means ± SEM (*n* = 8–15 rats per group). Key to statistics: *, **, *** = *p* < 0.05, 0.01, 0.001, respectively, in comparison to saline rats; ^#^, ^###^ = *p* < 0.05, 0.001, respectively, in comparison to ShA rats; ^!!!^ = *p* < 0.001, within group comparison to withdrawal day 31

Several groups of investigators have reported that rats undergoing self-administration of several types of drugs including opioids such as heroin exhibit increased active lever pressing in the presence of SA cues after long periods of forced withdrawal [15, 36–39]. To explore the possibility that rats that self-administered oxycodone behaved similarly, we compared the total amount of oxycodone consumption per rat (mg/kg, Fig. 1a) in relation to cue-induced active lever presses for each group on withdrawal days 5, 10, 19, and 31 (Fig. 2b) under conditions during which the rats did not obtain any contingent oxycodone infusions. An ANOVA consisting of group, extinction day, and their interactions found statistically significant effects of group (*F*_(3,34)_ = 14.88, *p* < 0.05), extinction day (*F*_(3,102)_ = 23.14, *p* < 0.05) and their interactions (*F*_(9,102)_ = 4.403, *p* < 0.05). Bonferroni post hoc tests showed both LgA-H (*p* < 0.0001) and LgA-L (*p* = 0.035) rats showed increased lever responses compared to saline rats, while LgA-H rats showed higher lever pressing in comparison to LgA-L rats (*p* = 0.05). Both LgA-L (*F*_(3, 20)_ = 4.87, *p* < 0.01) and LgA-H (*F*_(3, 32)_ = 5.38, *p* < 0.01) groups demonstrated higher number of lever presses at withdrawal day 31 in comparison to other withdrawal days (Figs. 2b). Similar statistical analyses revealed that the number of lever presses at withdrawal day 31 in the LgA phenotypes were significantly higher in comparison to those of the saline and ShA groups (*F*_(3, 34)_ = 16.86, *p* < 0.001) (Fig. 2b). There were no significant changes in lever pressing by the saline (*F*_(3, 28)_ = 1.35, *p* = 0.28) or ShA (*F*_(3, 56)_ = 1.14, *p* = 0.34) groups during the 31 days of observation (Fig. 2b). Regression analyses revealed positive correlations between amount of oxycodone consumed per individual animals and extinction day 5 (Fig. 2c, *p* = 0.0002) or extinction day 31 (Fig. 2 d, *p* < 0.0001).

### Forced Abstinence Is Associated with Downregulation of Mu Opioid Receptors in the Dorsal Striatum of LgA-L and LgA-H, but Not ShA, Rats

**Fig. 3:**
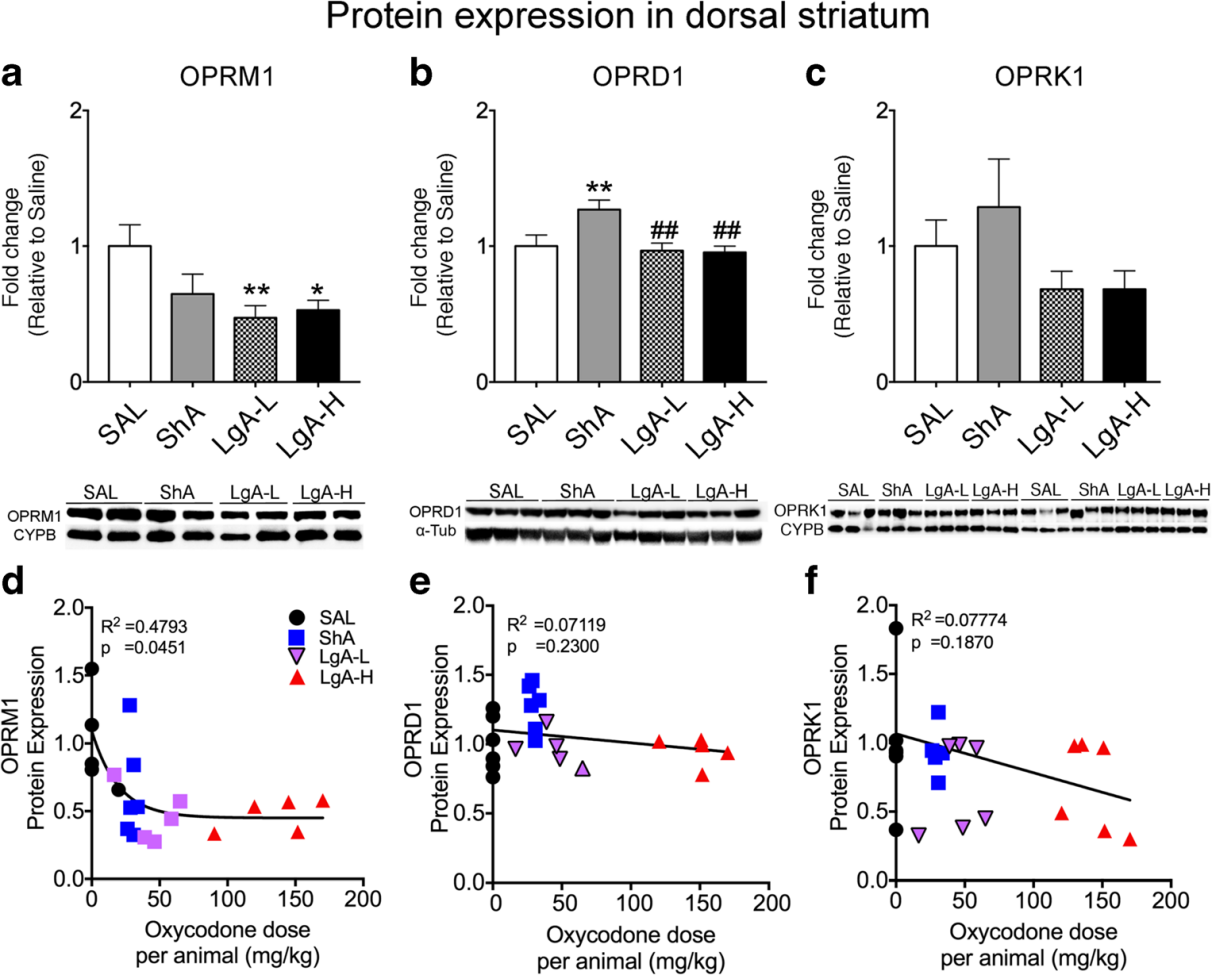
Mu receptor protein levels are decreased in the dorsal striatum of LgA rats after a month of forced abstinence. **a**–**c** Quantification of protein expression and representative images of Western blots showing levels of mu (OPRM1), delta (OPRD1), and kappa (OPRK1) proteins in rat striata. **a** LgA-L and LgA-H show decreased striatal OPRM1 protein levels. **b** Striatal OPRD1 protein levels show significant increases in the ShA rats. **c** Striatal OPRK1 protein levels were not significantly affected in any of the groups. **d** OPRM1 protein expression shows negative correlation to individual oxycodone intake. **e** OPRD1 and **f** OPRK1 protein expression show no significant relationship to drug intake. For quantitative Western blot analysis, the bands were normalized to cyclophilin B (CYPB) or α-tubulin (α-Tub). The values in the bar graphs represent means ± SEM (*n* = 5–6 rats per group). Key to statistics: *, **, *p* < 0.05, 0.01, respectively, in comparison to saline rats; ^##^*p* < 0.05 in comparison to ShA rats

Incubation of drug seeking is accompanied by molecular and biochemical adaptations in various brain regions [15, 37, 40]. However, a search of the literature did not identify any similar studies of oxycodone craving. Therefore, to identify potential biochemical changes in opioid receptor proteins that might have occurred following oxycodone SA and prolonged abstinence, we performed Western blot analyses using antibodies specific for mu (OPRM1), delta (OPRD1), and kappa (OPRK1) opioid receptors and measured their expression in the dorsal striatum and hippocampus, brain regions previously suggested to be involved in drug craving [15, 37, 40, 41]. In the striatum, there were statistically significant effects of groups (*F*_(3, 18)_ = 3.49, *p* = 0.0373). Post hoc comparisons revealed that both LgA-L (*p* = 0.0092) and LgA-H rats (*p* = 0.0142) exhibited significant decreased expression of OPRM1 compared to saline (Figs. 3a). Moreover, non-linear regression analysis showed that there was a correlation between the OPRM1 and oxycodone dose per rat (Fig. 3 d, *p* = 0.0451). In contrast, ShA rats exhibited increased OPRD1 protein levels compared to other groups (*F*_(3, 18)_ = 5.106, *p* < 0.01; Fig. 3 b, e). We found no significant changes in striatal OPRD1 protein expression in the LgA-L (*p* = 0.7298) or LgA-H (*p* = 0.6384) groups compared to saline (Fig. 3e). Linear regression analysis showed no relationship between OPRD1 and drug (Fig. 3e; *p* = 0.2300). Moreover, there were no significant changes in striatal OPRK1 protein levels (*F*_(3, 20)_ = 1.71, *p* = 0.20; Fig. 3c) after oxycodone SA and withdrawal. There was also no significant correlation between OPRK1 and oxycodone intake (Fig. 3f, *p* = 0.1870).

**Fig. 4:**
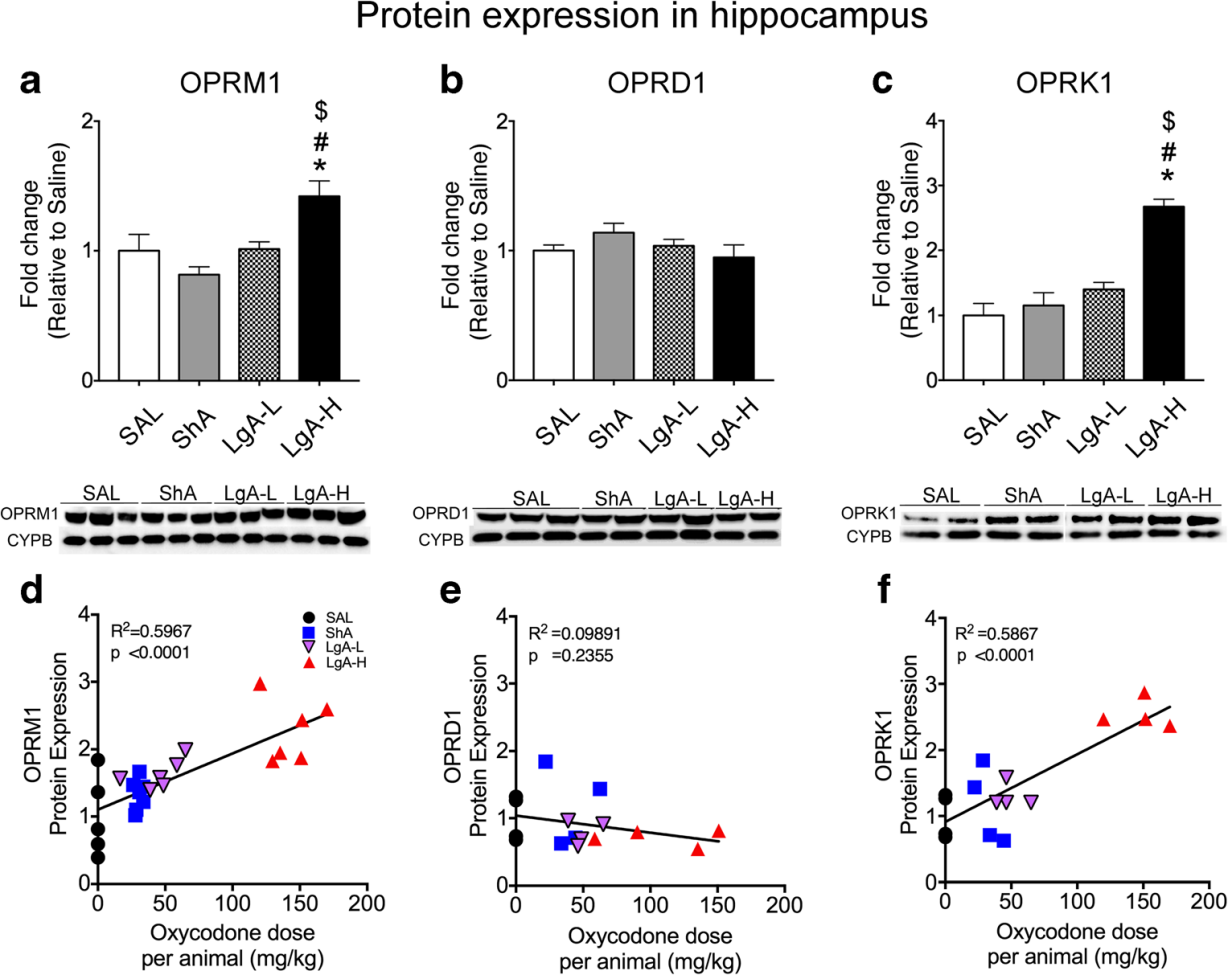
Mu and kappa protein levels are increased in the hippocampus of LgA-H rats after 1 month of forced abstinence and incubated behaviors. **a**, **b**, **c** Quantitative measures and representative images showing Western blot analyses for mu (OPRM1), delta (OPRD1), and kappa (OPRK1) receptor proteins, respectively, in the rat hippocampus. LgA-H rats show increased OPRM1 (**a**) and OPRK1 (**c**) receptor protein expression after a month of forced abstinence. **b** There were no significant changes in OPRD1 receptor protein levels. Individual oxycodone intake showed positive correlation to **d** OPRM1 and **f** OPRK1 protein levels in the hippocampus. **e** There was no significant relationship between the OPRD1 and oxycodone intake. For quantification, Western blotting for OPRM1, OPRD1, and OPRK1 proteins were normalized to cyclophilin B (CYPB) and then analyzed. The values represent means ± SEM (*n* = 5–6 rats per group). Note the differences in scales on the *Y*-axis. Key to statistics: *p < 0.05 in comparison to saline rats, ^#^*p* < 0.05 in comparison to ShA rats; ^$^*p* < 0.05 in comparison to LgA-L

In the hippocampus, there were statistically significant effects of oxycodone group on OPRM1 protein levels (Fig. 4 a, *F*_(3, 20)_ = 7.07, *p* < 0.002). Post hoc comparisons revealed that LgA-H rats had increased expression of hippocampal OPRM1 proteins relative to saline (*p* = 0.0057), ShA (*p* = 0.0002), and LgA-L (*p* = 0.0071) (Fig. 4a). Linear regression analysis revealed a positive correlation between OPRM1 protein levels and individual oxycodone doses (Fig. 4 d, *p* < 0.0001). There were also statistically significant effects of oxycodone group on hippocampal OPRK1 protein expression (*F*_(3, 16)_ = 14.37, *p* < 0.001). Post hoc comparisons showed that LgA-H rats also exhibited increased OPRK1 protein levels relative to saline (*p* < 0.0001), ShA (*p* < 0.0001), and LgA-L (*p* = 0.0005) Figs. 4c). There was also a significant correlation between OPRK1 protein expression and oxycodone intake (Fig. 4 f, *p* < 0.0001). There were no significant differences in hippocampal OPRD1 protein levels for any of the three oxycodone groups (*F*_(3, 33)_ = 1.31, *p* = 0.29; Fig. 4b) and the relationship between OPRD1 protein expression and oxycodone doses was not correlated (Fig. 4e, *p* = 0.2355).

### Differential Oxycodone Withdrawal-Associated Changes in Opioid Receptor mRNA Levels in the Striatum and Hippocampus

**Fig. 5:**
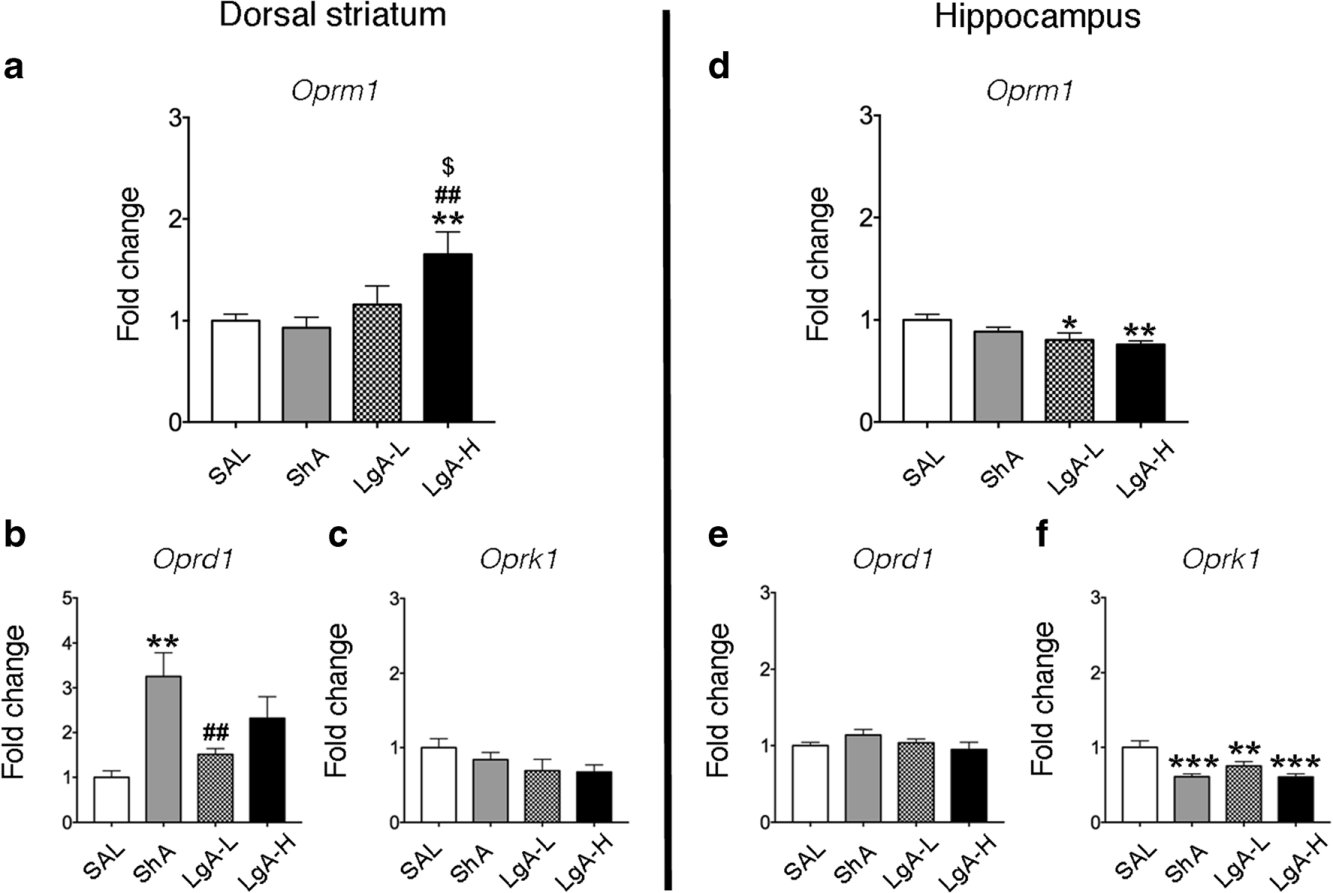
Differential changes in striatal and hippocampal mRNA expression after a month of forced abstinence from oxycodone SA. **a** Increased *Oprm1* receptor mRNA levels in the striatum of LgA-H rats. **b** Striatal *Oprd1* mRNA levels are increased in ShA rats. **c** Striatal *Oprk1* expression showed no significant changes. **d** Decreased hippocampal *Oprm1* receptor mRNA levels in LgA rats. **e** Hippocampal *Oprd1* mRNA levels show no significant changes. **f** Hippocampal *Oprk1* mRNA levels are decreased in all oxycodone groups. The values in the bar graphs represent means ± SEM (*n* = 5–12 animals per group). Note the differences in scales on the *Y*-axis. Key to statistics: *, **, *** = *p* < 0.05, 0.01, 0.001, respectively, in comparison to saline rats; ^##^*p* < 0.01 in comparison to ShA rats; ^$^*p* < 0.05 in comparison to LgA-L

To test if there were changes in opioid receptor mRNA levels corresponding to the alterations in protein levels, we used quantitative PCR to measure their mRNA levels in the same brain structures. There were significant effects of oxycodone group on striatal *Oprm1* mRNA levels (*F*_(3, 28)_ = 4.86; *p* = 0.0075, Fig. 5a). Fisher’s post hoc comparisons revealed that striatal *Oprm1* mRNA expression was significantly increased in the LgA-H phenotype compared to the other three groups: saline (*p* = 0.0072), ShA (*p* = 0.0011), and LgA-L (*p* = 0.0415). There were also significant effects of oxycodone group on hippocampal *Oprm1* mRNA levels (*F*_(3, 34)_ = 3.775, *p* = 0.0193, Fig. 5d), with transcript levels being significantly decreased in the LgA-L (*p* = 0.0260) and LgA-H (*p* = 0.0031) phenotypes in comparison to the control group. Consistent with protein data (Fig. 3b), there was a main effect of oxycodone group on striatal *Oprd1* mRNA (*F*_(3, 28)_ = 5.20, *p* = 0.005, Fig. 5b), with post hoc analysis showing increased delta transcripts only in the ShA group in comparison to the saline (*p* = 0.001) and LgA-L (*p* = 0.0117) groups. However, there were no significant changes in hippocampal *Oprd1* expression (*F*_(3, 33)_ = 1.314, *p* = 0.286, Fig. 5e). Finally, there were no significant changes in striatal *Oprk1* mRNA levels (*F*_(3, 28)_ = 1.646, *p* = 0.201, Fig. 5c) whereas *Oprk1* transcript expression was significantly decreased in the hippocampus in the three oxycodone groups in comparison to control (*F*_(3, 33)_ = 11.06, *p* < 0.001, Fig. 5f), further supporting the notion that oxycodone does also interact with kappa opioid receptors [32].

## Discussion

Oxycodone addiction and its associated complications have contributed immensely to the present public health crisis that is accompanied by an increase in opioid-related deaths around the USA [42, 43]. In the present study, we used oxycodone SA to identify potential biochemical and molecular markers that might serve as potential therapeutic targets. Our main findings are that long-access to oxycodone is accompanied by escalation of drug taking and incubation of oxycodone seeking after a month of drug withdrawal. These observations are consistent with an earlier report that described escalation of oxycodone intake by rats given 6-h [16] or 12-h daily access to oxycodone [18]. Mice, given a 4-h access to oxycodone, also escalated their intake of the drug [44]. Unfortunately, because these studies [16, 18, 44] had not reported on the behavioral effects of protracted withdrawal, it is difficult to come to any conclusion about incubation of oxycodone craving between our study and theirs. Our biochemical and molecular measures revealed, in addition, that mu, delta, and kappa receptor protein and mRNA levels were differentially impacted in the striatum and hippocampus of rats that had undergone protracted abstinence following various doses of oxycodone intake, with there being some differences even in rats that were exposed to oxycodone via the long-access paradigm. These differential regional responses are consistent with previous papers documenting brain regional effects of various drugs during different stages of addiction and withdrawal [24, 40, 45, 46].

It is of note that restricting oxycodone access to 3-h of exposure led to the rats not showing any escalation of drug intake. The present observations are consistent, in part, with an earlier study that showed restricted exposure (1-h exposure) to opioids including oxycodone did not show escalation of drug [18]. It is, therefore, possible to suggest that limited use of oxycodone in clinically controlled situations is advisable. It is also of interest to point out that the LgA rats that showed the highest oxycodone drug consumption (LgA-H) presented drug-seeking behavior earlier than the LgA-L rats. In particular, the LgA-H group started showing greater oxycodone seeking at withdrawal day 5 (see Fig. 2). If these results can be converted to human situations, they suggest that there needs to be even closer clinical observations of patients who use larger doses of oxycodone.

Our behavioral studies are also of interest because animals, given 6–9 h of daily access to oxycodone during the last 3 weeks of the study, fell into two distinct phenotypes, with some rats (LgA-H) taking very large quantities of the drug (see Figs. 1C and 2A). These results may be clinically relevant since not all humans who are exposed to oxycodone become addicted to large amounts of the drug. These behavioral observations are therefore consistent with the proposal that mammals may be predisposed to taking different amounts of drugs based on different individual hedonic set points [47] that are likely regulated by individual genetic makeups or predispositions.

Our observations that LgA-L and LgA-H rats show similar decreases in striatal mu receptors and similar incubation of oxycodone seeking suggest that striatal opioid receptor mechanisms might constitute pieces of the biochemical cascades that drive behavioral incubation in rodents and/or relapses in humans addicted to opioid drugs. This suggestion is supported by reports demonstrating a significant role of the dorsal striatum in cue-induced drug seeking [21, 48]. It could be argued, nevertheless, that observations of decreased expression of striatal mu opioid receptors might be due to oxycodone-induced motoric changes; however, this is not a tenable explanation because chronic use of opioids has very little consequences on locomotor activity [49, 50]. Importantly, even in the case of a psychostimulant such as cocaine, drug self-administration, and locomotor activity were not correlated [51], thus suggesting different neural substrates for these two behaviors. Moreover, our proposed mechanism is supported by the report that mu opioid agonists can be useful in the treatment of some symptoms of opioid addiction in humans [52]. Furthermore, the recent report that antagonism can block context-induced re-instatement provides additional support for this notion [16]. Nevertheless, this idea will need to be systematically tested in models of opioid addiction.

In contrast to the downregulation of mu receptor protein in the striatum of the LgA-H rats, there was significant upregulation of striatal *Oprm1* mRNA expression in those rats. This discrepancy between mRNA and protein levels in the LgA-H group indicates that decreased striatal OPRM1 expression was not secondary to changes in mRNA translation. In fact, in addition to mRNA translation, membrane protein abundance can be regulated by other factors including protein stability, degradation, and internalization [53]. Importantly, exposure of opioid receptors to their agonists can also cause desensitization of their receptors via similar mechanisms [54]. Thus, it is not far-fetched to suggest the possibility that the increases in *Oprm1* mRNA expression observed in the LgA-H rats might not have been sufficient to counteract the downregulation of OPRM1 protein expression observed after repeated exposure to oxycodone. Protein stability, degradation, and internalization might have promoted enhanced downregulation of membrane protein expression in the presence of high oxycodone doses. Interestingly, we also observed dissociation between mRNA and protein levels of mu and kappa opioid receptors in the hippocampus in the LgA-H group (compare data in Figs. 4 and 5). Specifically, there were increases in mu and kappa opioid protein levels in the hippocampus of LgA-H rats whereas there were decreases in hippocampal *Oprm1* and *Oprk1* mRNA levels in these rats. Our findings are consistent with previous demonstrations that the opioid antagonist, naltrexone, up-regulated mu opioid receptor protein expression [55, 56] without altering its mRNA expression [55, 57]. When taken together, these observations indicate that adaptive changes in the transcription of mu opioid receptors may not play a substantial role in regulating their functions in models of tolerance or addiction.

Although not of specific relevance to the actions of oxycodone, it needs to be noted that the observed dissociation between mRNA and protein levels for the mu and kappa opioid receptors after oxycodone SA and withdrawal are consistent with similar dissociation of BDNF mRNA/protein expression after self-administration of other drugs of abuse including cocaine and methamphetamine [37, 58, 59]. These findings suggest that abusable drugs can impact protein and gene expression differentially. Importantly, our observations of dissociation of mRNA/protein expression after oxycodone SA and withdrawal are consistent, in general, with findings of low levels of correlations between mRNA and protein levels detected in various biological systems including the brain [60–63].

Opioid addicts have been reported to suffer from neuropsychiatric signs and symptoms including affective disturbances [64–66]. Some of these psychiatric symptoms may be attributable to altered opioid dynorphin/kappa neurotransmission because kappa antagonism has antidepressant efficacy [67–69]. Indeed, our observations of significant increases in mu and kappa opioid receptor protein expression in the hippocampus of only LgA-H rats suggest that there might be increased mu and kappa receptor-mediated signaling in the hippocampus of those rats. These observations provide further support for our decision to dichotomize the LgA rats into LgA-L and LgA-H based on their actual oxycodone infusions. The identified increases in mu receptor protein expression in the hippocampus in the LgA-H in comparison to the LgA-L rats contrast with the similar decreases observed in the striatum of the LgA-L and LgA-H rats that showed similar increases in lever pressing after protracted withdrawal, suggesting that striatal mu opioid-regulated mechanisms might be more involved in the manifestation of incubation of oxycodone seeking. Our data also appear to be consistent with clinical observations that humans who showed greater escalation of their intake of opioid drugs may be more prone to suffer from some psychiatric diatheses [64–66]. These results further suggest that hippocampal mechanisms may be more relevant to psychiatric sequelae of high-dose opioid abuse. These statements are also consistent with the demonstration that stimulation of kappa receptors can attenuate dopamine neurotransmission within the mesolimbic reward pathway [70]. Thus, it is not far-fetched to suggest that kappa signaling-induced decreases in hippocampal DA neurotransmission might serve as partial substrates for the affective diatheses observed in some humans who abuse large amounts of opioid medications [64–66]. The veracity of these ideas will need to be tested in both preclinical models and clinical settings.

It is also of singular interest that only ShA rats that had not exhibited increased oxycodone seeking behaviors during protracted abstinence (see Fig. 2b) showed increased striatal delta opioid receptor mRNA and protein levels (see Figs. 3b and 5b). As discussed in the results section, there were no significant changes in striatal delta opioid receptors in the brains of LgA-L and LgA-H rats that had shown time-dependent increases in oxycodone seeking during drug withdrawal. These observations suggest that increased expression of opioid receptor might serve, in part, to suppress withdrawal-induced incubation by a yet unknown process that might involve cholinergic interneurons that are the major source of striatal delta opioid receptors [71]. This idea is supported by the fact that stimulation of delta opioid receptors inhibits acetylcholine release in the striatum [72, 73]. Although this idea will need to be tested, it seems plausible because perturbations of both muscarinic and nicotinic acetylcholine receptors impact cue-induced reward seeking [74].

In summary, exposure to extended oxycodone SA is accompanied by escalation of drug intake and incubation of drug seeking after several days of forced abstinence. This is also the first study to shown that incubated oxycodone seeking behaviors are accompanied by similar changes in striatal mu opioid receptor protein levels in the dorsal striata of LgA-L and LgA-H rats, suggesting that these changes might, in part, serve as substrates for behavioral incubation of oxycodone seeking in rats. In contrast, only the LgA-H rats showed increased expression of hippocampal mu and kappa receptors indicating that large doses of oxycodone may be necessary to cause changes in hippocampus-dependent learning and memory processes that might trigger psychiatric diatheses in humans addicted to opioids. Finally, regional effects of oxycodone on the brain need to be taken into consideration when planning experiments that focus on the development of therapeutic interventions for oxycodone addiction.

## Electronic supplementary material

S1. **ShA rats take higher oxycodone infusions during first hours of daily drug self-administration.** (A) Rats show higher infusions during the second hour compared to the first hour of training day 1. (B-E) In the following training days rats took more oxycodone during the first hour in comparison to the second and third hour. The values in the line graphs represent means ± SEM (*n* = 15). Key to statistics: **, *** = *p* < 0.01, 0.001, respectively, in comparison to first or second hour as described in the figure; $$, $$$ = *p* < 0.01, 0.001, respectively in comparison to third hour. (PNG 123 kb)

High resolution image (TIF 9486 kb)

S2. **LgA-L rats take more oxycodone infusions during first hours of the three 3-h daily sessions of drug self-administration.** (A) There were no significant differences in hourly intake during training day 1. (B-E) In subsequent days, the rats took more oxycodone during the first hour in comparison to the second and third hour of each three 3-h daily sessions. The number of oxycodone infusions during the second hour was also higher, for the most part, in comparison to the third hour of each 3-h session. The values in the line graphs represent means ± SEM (*n* = 6 animals per group). Key to statistics: **, *** = *p* < 0.01, 0.001, respectively, in comparison to third or ninth hour as described in the figure; #, ## = *p* < 0.05, 0.01, respectively in comparison to second, fourth, or eighth hour; $$, $$$ = *p* < 0.01, 0.001, respectively in comparison to third or ninth hour. (PNG 164 kb)

High resolution image (TIF 15497 kb)

S3. **LgA-H rats take more oxycodone infusions during first hours of the three 3-h daily sessions of drug self-administration.** (A) There were no significant differences in hourly intake during the first training day. (B, D) The number of oxycodone infusions was higher in the first hour in comparison to the second or third hours of the first 3-h session. (C, D) Number of infusion during the second hour was also higher in comparison to infusions during the third hour. (C, E) During the second 3-h daily session, the number of infusions during the fourth hour (first hour of second 3-h session) was higher than the number during the sixth hour. (D, E) During the third 3-h session, the number of infusions during the seventh hour (first hour of the third 3-h session) was also greater than oxycodone infusions during the eighth and ninth hour. The values in the line graphs represent means ± SEM (*n* = 9 animals per group). Key to statistics: *, **, *** = *p* < 0.05, 0.01, 0.001, respectively, in comparison to third or ninth hour; #, ## = *p* < 0.05, 0.01, respectively, in comparison to second, fourth, or eighth hour; $$, $$$ = *p* < 0.01, 0.001, respectively, in comparison to third or ninth hour. (PNG 175 kb)

High resolution image (TIF 15484 kb)
