# Supplementary figures and images for: Molecular Adaptations in the Rat Dorsal Striatum and Hippocampus Following Abstinence-Induced Incubation of Drug Seeking After Escalated Oxycodone Self-Administration
Source: Mol Neurobiol. 2018 Aug 28;56(5):3603–15. doi: 10.1007/s12035-018-1318-z (PMC6477015; doi:10.1007/s12035-018-1318-z)

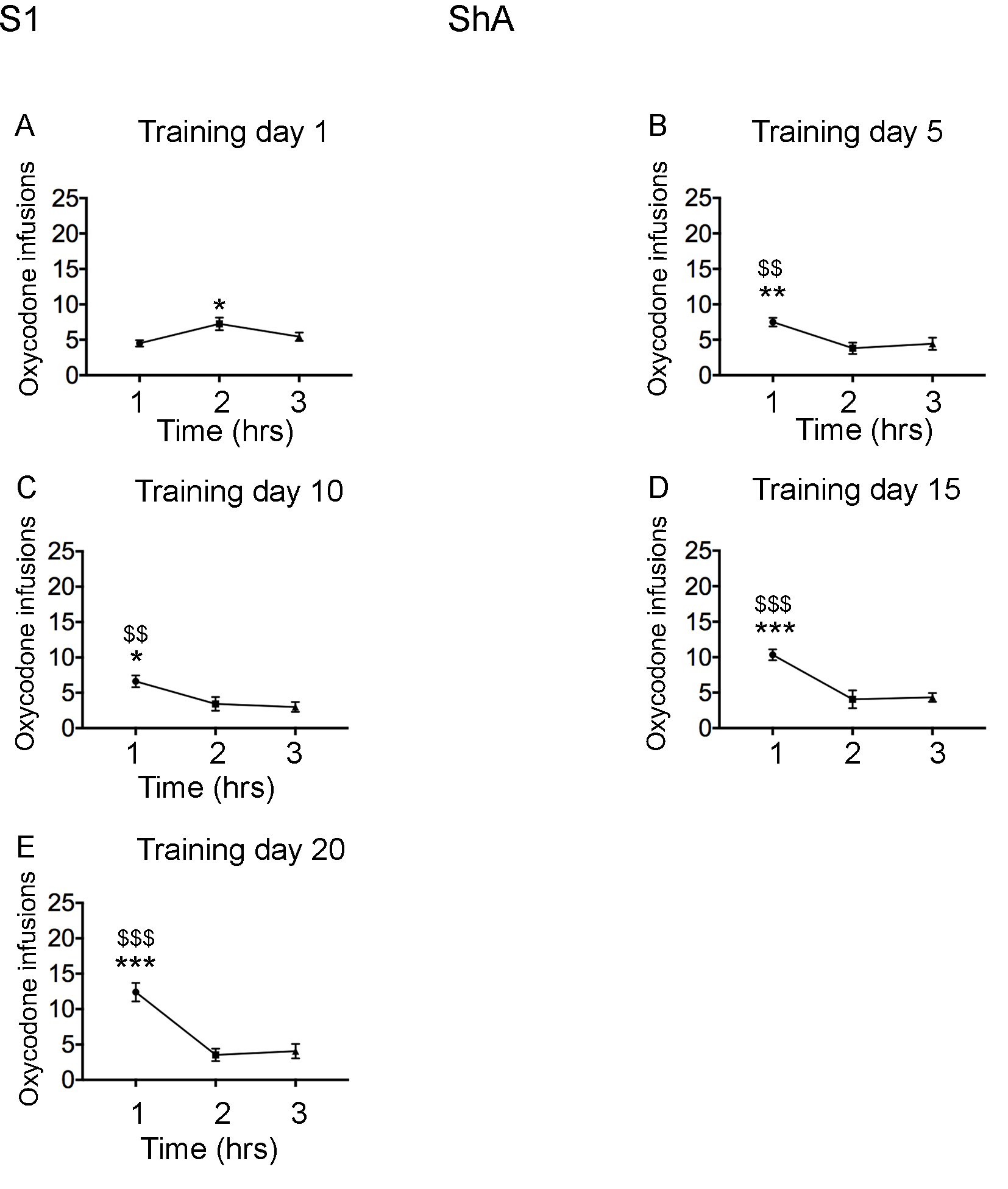

Supplement: Supplementary file 1 — ShA rats take higher oxycodone infusions during first hours of daily drug self-administration. (A) Rats show higher infusions during the second hour compared to the first hour of training day 1. (B-E) In the following training days rats took more oxycodone during the first hour in comparison to the second and third hour. The values in the line graphs represent means ± SEM (n = 15). Key to statistics: **, *** = p < 0.01, 0.001, respectively, in comparison to first or second hour as described in the figure; $$, $$$ = p < 0.01, 0.001, respectively in comparison to third hour. (PNG 123 kb) [file 12035_2018_1318_Fig6_ESM.png]

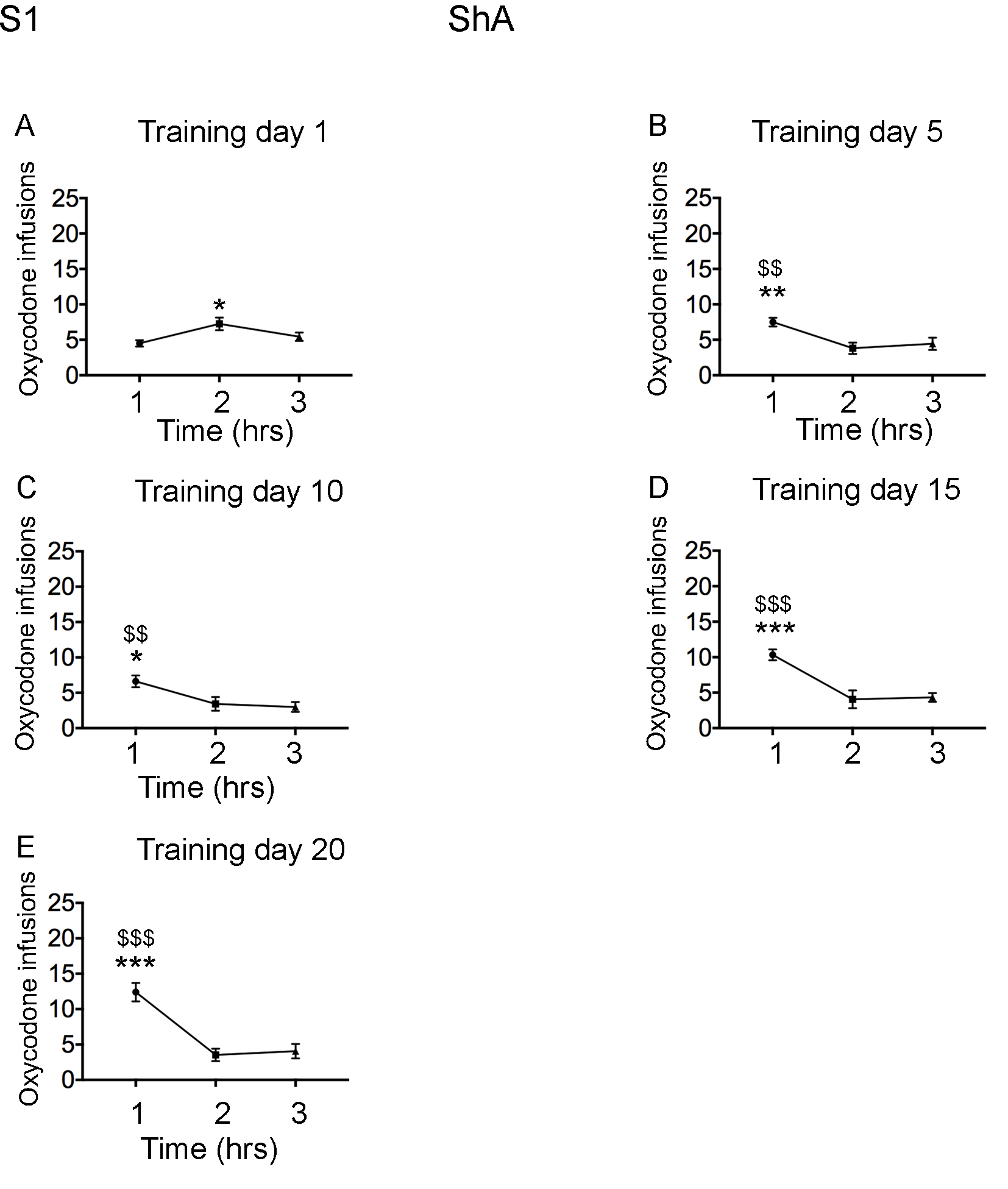

Supplement: Supplementary file 2 — High resolution image (TIF 9486 kb) [file 12035_2018_1318_MOESM1_ESM.tif]

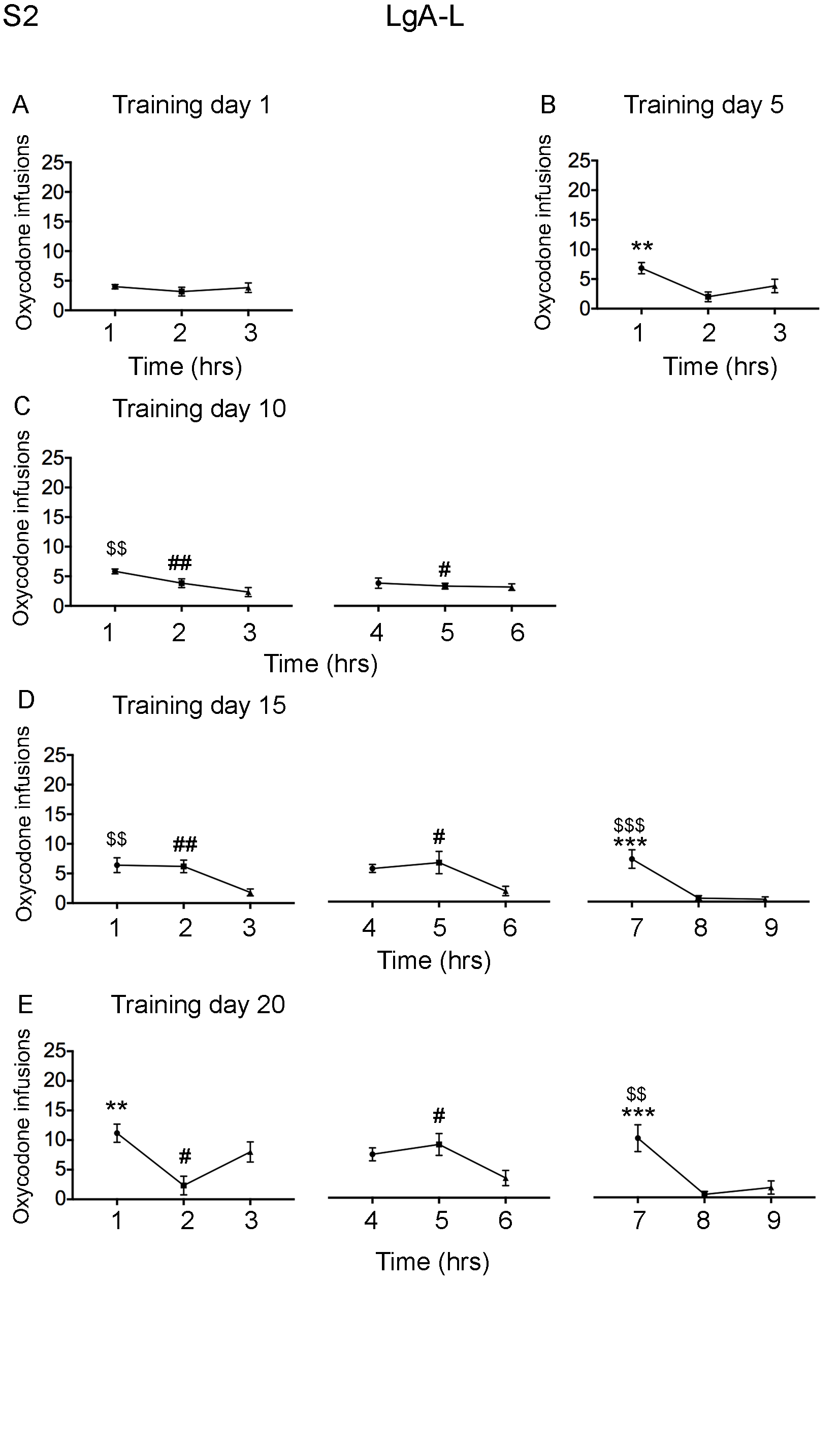

Supplement: Supplementary file 3 — LgA-L rats take more oxycodone infusions during first hours of the three 3-h daily sessions of drug self-administration. (A) There were no significant differences in hourly intake during training day 1. (B-E) In subsequent days, the rats took more oxycodone during the first hour in comparison to the second and third hour of each three 3-h daily sessions. The number of oxycodone infusions during the second hour was also higher, for the most part, in comparison to the third hour of each 3-h session. The values in the line graphs represent means ± SEM (n = 6 animals per group). Key to statistics: **, *** = p < 0.01, 0.001, respectively, in comparison to third or ninth hour as described in the figure; #, ## = p < 0.05, 0.01, respectively in comparison to second, fourth, or eighth hour; $$, $$$ = p < 0.01, 0.001, respectively in comparison to third or ninth hour. (PNG 164 kb) [file 12035_2018_1318_Fig7_ESM.png]

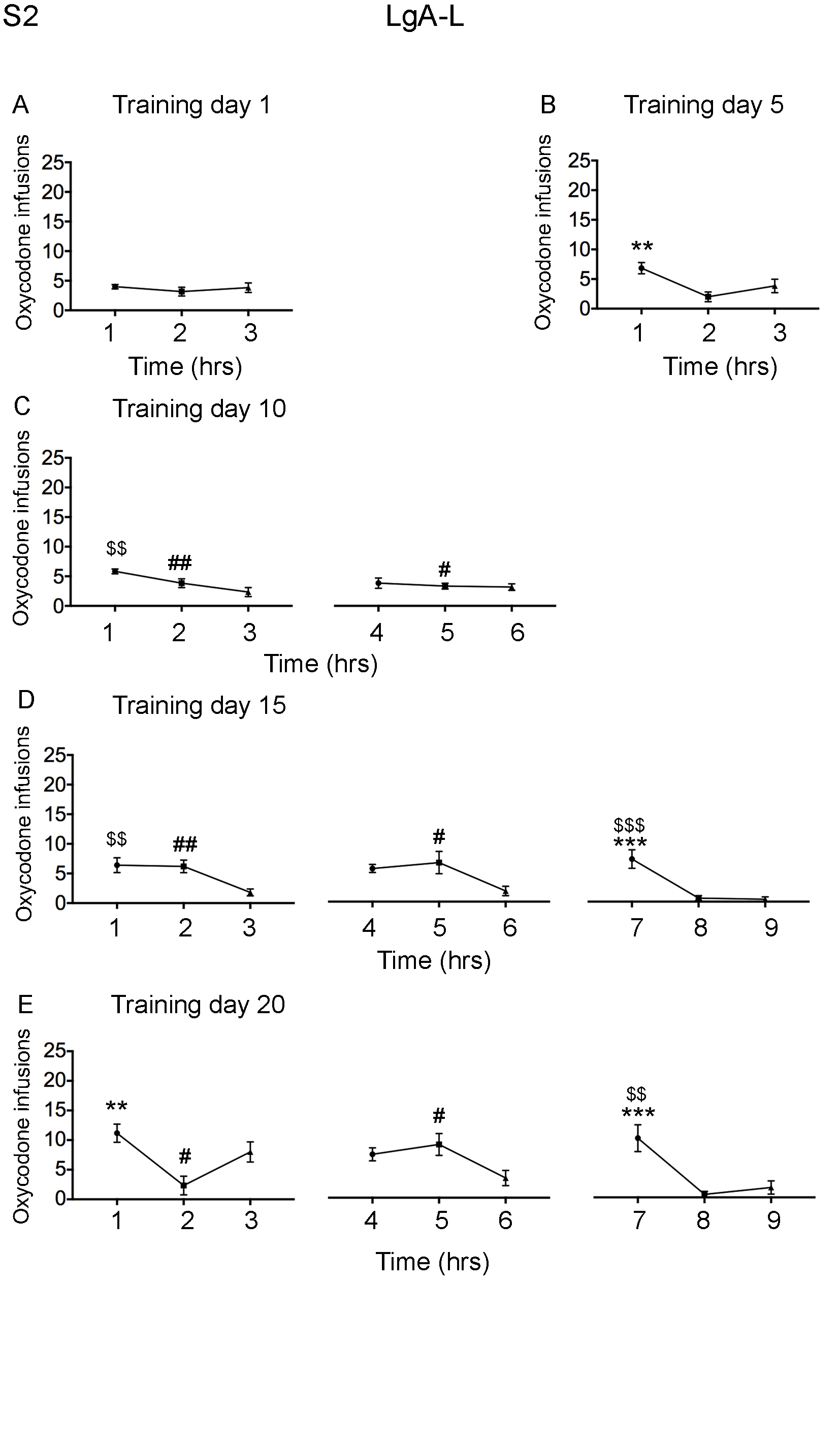

Supplement: Supplementary file 4 — High resolution image (TIF 15497 kb) [file 12035_2018_1318_MOESM2_ESM.tif]

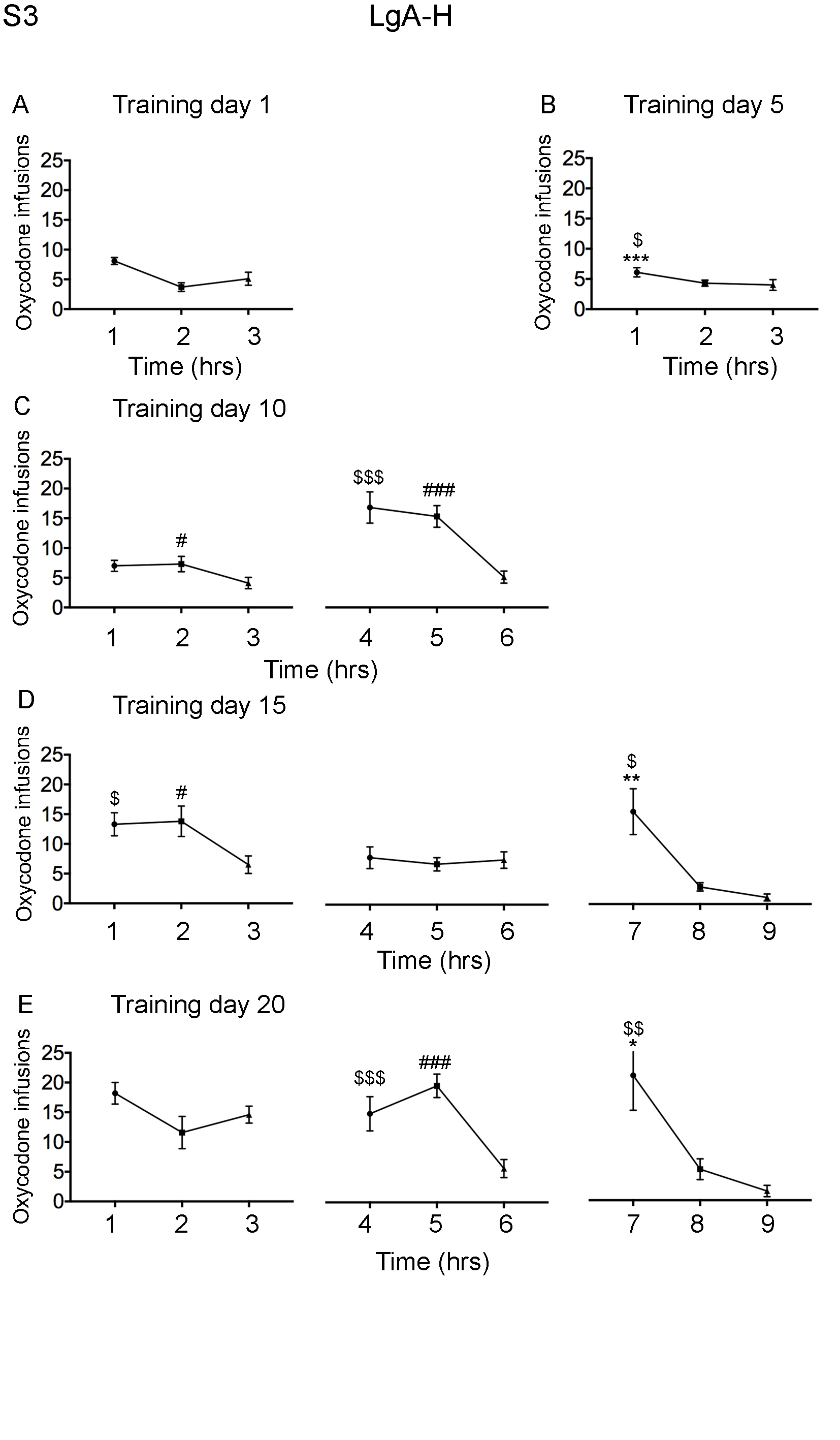

Supplement: Supplementary file 5 — LgA-H rats take more oxycodone infusions during first hours of the three 3-h daily sessions of drug self-administration. (A) There were no significant differences in hourly intake during the first training day. (B, D) The number of oxycodone infusions was higher in the first hour in comparison to the second or third hours of the first 3-h session. (C, D) Number of infusion during the second hour was also higher in comparison to infusions during the third hour. (C, E) During the second 3-h daily session, the number of infusions during the fourth hour (first hour of second 3-h session) was higher than the number during the sixth hour. (D, E) During the third 3-h session, the number of infusions during the seventh hour (first hour of the third 3-h session) was also greater than oxycodone infusions during the eighth and ninth hour. The values in the line graphs represent means ± SEM (n = 9 animals per group). Key to statistics: *, **, *** = p < 0.05, 0.01, 0.001, respectively, in comparison to third or ninth hour; #, ## = p < 0.05, 0.01, respectively, in comparison to second, fourth, or eighth hour; $$, $$$ = p < 0.01, 0.001, respectively, in comparison to third or ninth hour. (PNG 175 kb) [file 12035_2018_1318_Fig8_ESM.png]

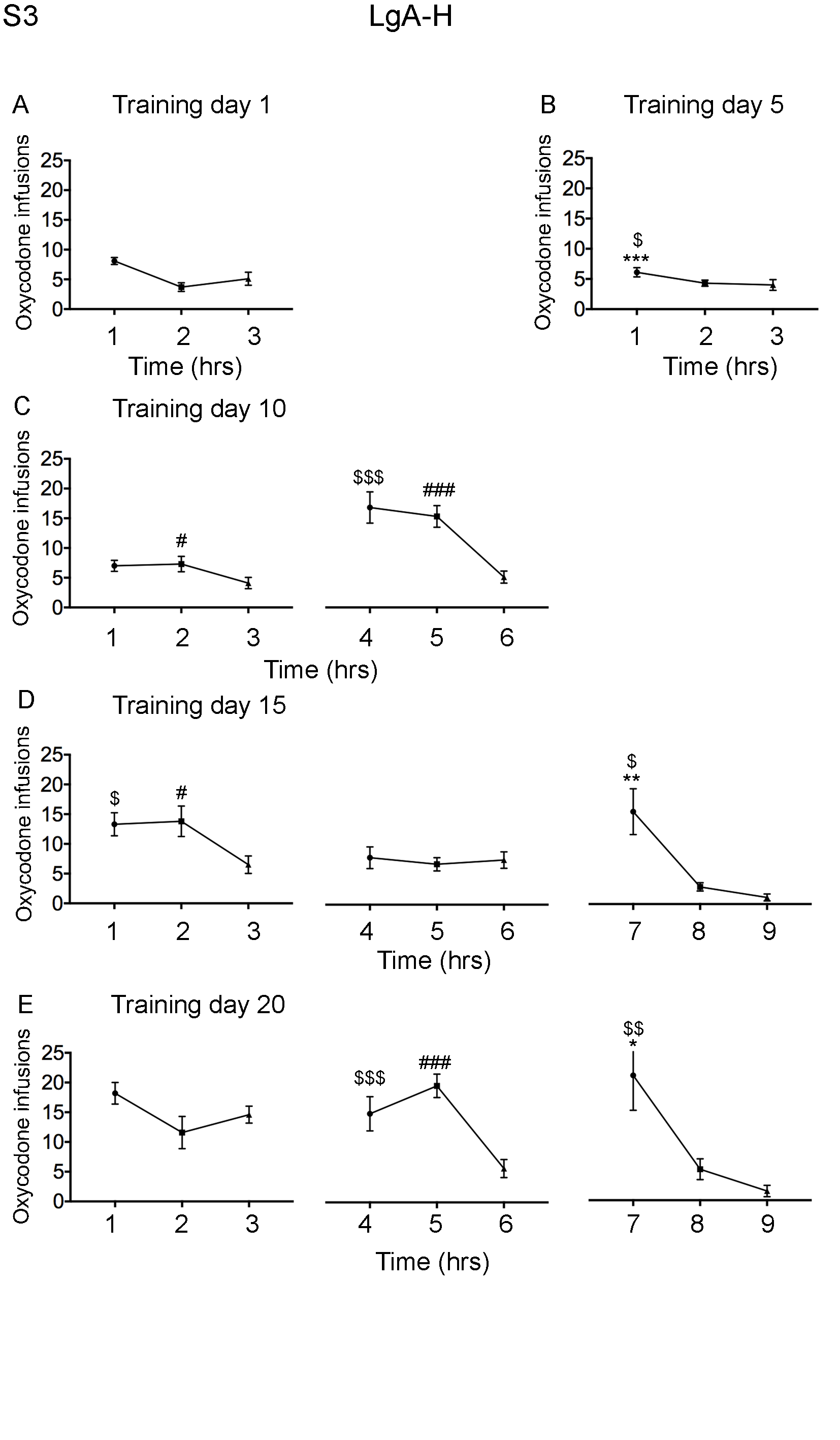

Supplement: Supplementary file 6 — High resolution image (TIF 15484 kb) [file 12035_2018_1318_MOESM3_ESM.tif]
